# *Bifidobacterium pseudocatenulatum*-Mediated Bile Acid Metabolism to Prevent Rheumatoid Arthritis via the Gut–Joint Axis

**DOI:** 10.3390/nu15020255

**Published:** 2023-01-04

**Authors:** Qing Zhao, Huan Ren, Nian Yang, Xuyang Xia, Qifeng Chen, Dingding Zhou, Zhaoqian Liu, Xiaoping Chen, Yao Chen, Weihua Huang, Honghao Zhou, Heng Xu, Wei Zhang

**Affiliations:** 1 Department of Clinical Pharmacology, Xiangya Hospital, Central South University, 87 Xiangya Road, Changsha 410008, China; 2 Hunan Key Laboratory of Pharmacogenetics, Institute of Clinical Pharmacology, Central South University, 110 Xiangya Road, Changsha 410078, China; 3 Engineering Research Center of Applied Technology of Pharmacogenomics, Ministry of Education, 110 Xiangya Road, Changsha 410078, China; 4 National Clinical Research Center for Geriatric Disorders, 87 Xiangya Road, Changsha 410008, China; 5 State Key Laboratory of Biotherapy and Cancer Center, West China Hospital, Sichuan University, Chengdu 610041, China; 6 Department of Laboratory Medicine, West China Hospital, Sichuan University, Chengdu 610041, China; 7 Department of Pathology, Xiangya Hospital, Central South University, 87 Xiangya Road, Changsha 410008, China

**Keywords:** *Bifidobacterium pseudocatenulatum*, rheumatoid arthritis, bile acid metabolism, gut–joint axis, Takeda G protein-coupled receptor 5, Th1/Th17

## Abstract

Early intervention in rheumatoid arthritis (RA) is critical for optimal treatment, but initiation of pharmacotherapy to prevent damage remains unsatisfactory currently. Manipulation of the gut microbiome and microbial metabolites can be effective in protecting against RA. Thus, probiotics can be utilized to explore new strategies for preventing joint damage. The aim of this study was to explore the metabolites and mechanisms by which *Bifidobacterium pseudocatenulatum* affects RA. Based on 16S rRNA sequencing and UPLC-MS/MS assays, we focused on bile acid (BA) metabolism. In a collagen-induced arthritis (CIA) mouse model, *B. pseudocatenulatum* prevented joint damage by protecting the intestinal barrier and reshaped gut microbial composition, thereby elevating bile salt hydrolase (BSH) enzyme activity and increasing the levels of unconjugated secondary BAs to suppress aberrant T-helper 1/17-type immune responses; however, these benefits were eliminated by the Takeda G protein-coupled receptor 5 (TGR5) antagonist SBI-115. The results suggested that a single bacterium, *B. pseudocatenulatum*, can prevent RA, indicating that prophylactic administration of probiotics may be an effective therapy.

## 1. Introduction

Rheumatoid arthritis (RA) is a common systemic inflammatory autoimmune disease that causes chronic synovitis and bone destruction [1]. Remission of RA is arguably the ultimate goal of anti-rheumatic therapy, and current initiating treatment with agents to block the progression of bone destruction during the pre-arthritic stages remains unsatisfactory [2,3]. Therefore, there is an exigent requirement to explore and substantiate affordable, effective and safer interventions to prevent and cure RA.

Much compelling evidence indicates that probiotics can be used to develop new strategies for the prevention and early treatment of RA, owing to their limited toxic effects [3,4,5]. Given the wide variety of probiotic species, it is important to identify not only the species of the probiotic to be used but also the specific strain. At the single-strain level, some probiotics, such as *Bifidobacterium* spp. And *Lactobacillus* spp., have been demonstrated to alleviate disease by interacting with the gut microbiota in animal and clinical studies [3,4]. *Bifidobacterium* is one of the most predominant commensal bacteria in the gut and has been shown to have a major ecological function in shaping the intestinal microbiome [6]. The beneficial effects of *Bifidobacterium* on specific diseases have been widely reported, with preclinical studies demonstrating its ability to minimize chronic inflammation and balance the immune response involved in autoimmune diseases [7,8,9]. In addition, *B. pseudocatenulatum* has been reported to inhibit inflammation, thereby reversing the adverse effects of diet-induced obesity, and is a promising probiotic that can modulate the gut–bone axis [10]. Despite these reports, knowledge of whether a single bacterium, more specifically *B. pseudocatenulatum*, as a probiotic can prevent or ameliorate disease is still limited, and the interactions involved in the gut–joint axis remain to be demonstrated in RA.

Bile salt hydrolase (BSH) hydrolyzes conjugated BAs in the intestine to form amino acids and de-conjugated Bas (e.g., LCA and DCA) and is mainly secreted by probiotic bacteria during growth and reproduction [11,12]. BAs, in turn, can reshape the composition of the gut microbiota by directly regulating bile metabolizing bacteria and BSH activity [13]. Bacteria with bile salt hydrolase (BSH) activity are widespread in the human or animal intestine, mainly in *Lactobacillus* spp., *Clostridium* spp., and *Bifidobacterium* spp. [14]. LCA and DCA are the most potent agonists of Takeda G protein-coupled receptor 5 (TGR5), a bile acid-activated membrane receptor that mediates the anti-inflammatory function of certain BAs [12]. Thus, the interaction between the gut microbiota and BAs is crucial for the regulation of the inflammatory response and the maintenance of immune homeostasis in autoimmune disease [15]. In particular, regulation of CD4+ T-cell differentiation is essential to maintain an optimal balance of CD4+ T-cell subsets to support immune homeostasis and prevent autoimmunity [16,17,18]. However, the role of gut microbiota-mediated changes in BA composition on the regulatory mechanisms of CD4+ T-cell to improve disease progression in RA has not been reported; hence the subject of the study presented here.

In this study, we tested the impact of probiotic intervention on BAs and gut microbiota composition. We uncover a critical role for *B. pseudocatenulatum* in the prevention of TGR5-mediated CD4+T cell-driven pathology development in an RA-like mouse model. Collectively, this work highlights the interplay between the probiotic, CD4+T cell immunity, and BA metabolism in the context of RA.

## 2. Materials and Methods

### 2.1. Human Cohorts

This study was performed in according to established ethical guidelines and was approved by the Research Ethics Committee of West China Hospital, Sichuan University (Chengdu, China). All subjects provided written informed consent prior to the start of the study.

Clinical cohort I recruited stool samples from 36 treatment-naïve new-onset RA patients (NORA) and 11 healthy family members, and the patient group was further divided into positive and negative groups based on the presence of anti-keratin antibody (AKA). Clinical characteristics are shown in Table S1.

Clinical cohort II recruited 40 well-established RA patients with disease-modifying antirheumatic drug (DMARD) treatment and 34 healthy individuals, which were further divided into DJ (deformed joints; *n* = 18) and NDJ (no deformed joint; 3 years after diagnosis, *n* = 22) groups based on the presence or absence of sequelae of joint damage after drug administration. Clinical characteristics are shown in Table S2. All samples were stored at −80 °C until further analysis.

Clinical cohort III analyzed 233 records of RA and 16,282 records of Healthy in GMrepo database (a curated database of human gut metagenomes), and 126 records of RA and 7066 records of Healthy were filtered for further analysis based on whether they expressed *B. pseudocatenulatum*.

### 2.2. Co-Culture Model

Intestinal epithelial IEC-6 cells and Caco-2 cells were purchased from ATCC (Manassas, VA, USA) and cultured at 37 °C, 5% CO_2_ in DMEM (Gibco, Grand Island, NY, USA) with 10% heat-inactivated fetal bovine serum (FBS, Gibco, Grand Island, NY, USA). Caco-2 cells are human clonal colon adenocarcinoma cells, structurally and functionally similar to differentiated small intestinal epithelial cells.

After apposition, cells were incubated for 6 h under lipopolysaccharide (LPS, 1 μg/mL, Sigma-Aldrich, St. Louis, MO, USA) stimulation to produce pro-inflammatory cytokines. Subsequently, a final concentration of 10^6^ CFU/mL of live bacterial suspension was added to the medium. Culture samples were collected after 6–8 h to detect the secretion of TNF-α, IFN-γ, and IL-17A.

### 2.3. Animal Experiments

Six-week-old male DBA/1J mice were purchased from Cavens Experimental Animal Co., Ltd. (Changzhou, China). All the experiment processes and animal care were carried out in accordance with the regulations of the Animal Ethics Committee of Xiangya Hospital, Central South University (Changsha, China). After adaptation, all mice were given antibiotic treatment (ABX) for 7 days to deplete the gut microbiota using drinking water containing 1 g/L neomycin, 1 g/L ampicillin, 1 g/L metronidazole, and 0.5 g/L vancomycin [19]. Then, the different experimental groups were established.

One schedule of the experiment: daily oral administration with 0.2 mL *B. pseudocatenulatum* cocktail (OD600 of 1.0, corresponding to 1 × 10^9^ CFU) was given to the prevention group (Bi.pse + CIA, *n* = 6) for 21 days starting on day 7 before the first immunization. The treatment group (CIA + Bi.pse, *n* = 6) was given *B. pseudocatenulatum* cocktail after the booster immunization for 21 days through daily oral gavage. Meanwhile, controls and CIA mice were treated with saline through oral gavage during the whole trial.

Another schedule of the experiment: daily oral administration with 0.2 mL *B. pseudocatenulatum* cocktail was given for 21 days starting on day 7 before the first immunization. After the booster immunization, deoxycholic acid (DCA, 50 mg/kg/day), lithocholic acid (LCA, 50 mg/kg/day), INT-777 (a semi-synthetic TGR5 agonist, 80 mg/kg/day), and SBI-115 (a TGR5 antagonist, 80 mg/kg/day, MedChemExpress, Monmouth Junction, NJ, USA) were given for 28 days.

On day 50 of the experiment, all mice were deeply anesthetized with 4% isoflurane (Abbott, Cham, Switzerland) by inhalation, whole blood was collected in test tubes by removing the eyeball, and mice were sacrificed quickly by cervical dislocation, then ileal tissue and paw samples were collected from each mouse.

### 2.4. Flow Cytometry

Cells from blood samples were characterized based on cell surface markers using fluorescence-activated cell sorting (FACS) analyses. The cells were stained with different fluorescently labeled monoclonal antibodies (mAb). In brief, 5 × 10^5^ cells suspended in 100 μL of PBS with brefeldin A (Biolegend 423303) were mixed with 10 μL of 1640 medium and were incubated in the dark at 37 °C for 5 h. The cell pellets were washed twice with PBS containing 2% BSA and were resuspended in PBS. Subsequent flow cytometry analysis was done immediately using the mouse anti-human IL-17A-PE mAb (Biolegend 506904), IFN-γ-BV421 mAb (Biolegend 505830), CD3-APC mAb (Biolegend 155606), CD8-PECY7 mAb (Biolegend 980910), CD4-FITC mAb (Biolegend 100529), CD25-PECY7 mAb (Biolegend 102016), FOXP3-AF647 mAb (Biolegend 320214), and CD45-APC-CY7 mAb (Biolegend 103154), Live-dead (Biolegend 423101).

The fluorescence intensity of the cells was evaluated by EPICS-XL flow cytometer (BD Biosciences, Franklin Lakes, NJ, USA). FlowJo V10 was used to further analyze the levels of Th1, Th17 and Treg cells. The following markers were used to identify different immune cell subsets: CD4+IFN-γ+ for Th1, CD4+IL-17A+ for Th17 cells, CD4+CD25+foxp3+ for Treg.

### 2.5. Quantification of Bile Acids in Feces

To quantify bile acids in feces, 20 mg mice stool samples were weighed, added to 1 mL water-methanol (5:5 *v*/*v*), vortex mixed, sonicated for 30 s and centrifuged, and 100 μL supernatants were collected and dried under a gentle stream of nitrogen. The dried metabolite residues were dissolved in 200 μL of 50% methonal and 10 μL was injected for quantification by ultra-performance liquid chromatography coupled with tandem mass spectrometry (UPLC-MS/MS, Ab SCIEX, USA, Triple Quad 6500+). A ACQUITY UPLC HSST3 C18 analytical column (2.1 mm * 100 mm, 1.8 mm) was used for chromatographic separation with the mobile phases being a mixture of 0.1% formic acid and 5 mM ammonium acetate in water (A) and methanol (B). The column temperature was 50 °C and the flow rate was 0.2 mL min^−1^. The gradient was set as follows: 58–60% B at 0–10 min, 60–66% B at 10–15 min, 66–70% B at 15–20 min, 70–80% B at 20–25 min, 80–90% B at 25–30 min, 90% B at 30–32 min, 90–50% B at 32–36 min, and 50% B at 36–40 min. Thirty-eight BAs were involved in the quantification by UPLC-MS/MS. Concentrations of the detected BAs were calculated with internal standard calibration from the linearly regressed standard calibration curves of individual BAs. The lower limit of quantification was 0.08 nmol/mg for all the BAs.

### 2.6. Statistical Analysis

The data were analyzed using the Graphpad Prism 9.0 software (Graphpad Software Inc., San Diego, CA, USA). Data are shown as means ± SEM or mean ± SD, and the *p*-values were calculated by unpaired t-tests, one-way analysis of variance (ANOVA) and post-hoc Dunnett’s test. Corrected *p*-values were used to account for multiple testing. Statistical significance is indicated by asterisks (*): * *p* < 0.05, ** *p* < 0.01, *** *p* < 0.001, **** *p* < 0.001, ns, non-significant. All reported analyses were considered significant at *p* < 0.05.

## 3. Results

### 3.1. Bifidobacterium Abundance Was Negatively Correlated with Indicators of Disease Activity

**Figure 1:**
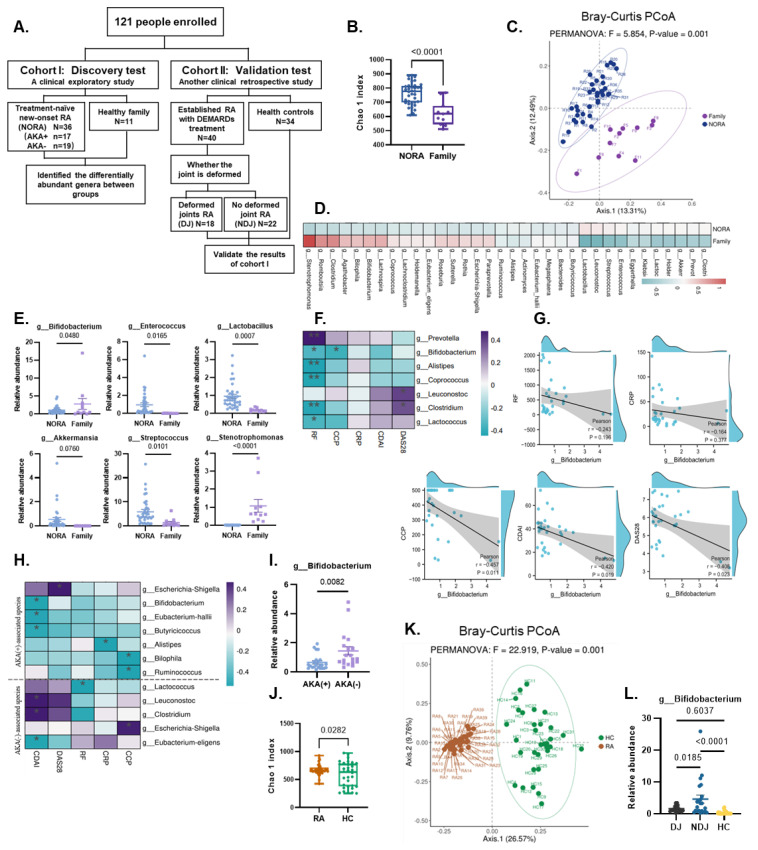
Gut microbial composition at the genus level and clinical relevance in RA patients and healthy controls of two cohorts. (**A**) A flow diagram depicting the enrollment and analysis of RA patients and healthy individuals. (**B**) Chao 1 index of the NORA cohort was significantly higher in RA patients compared to their relatives (*p* < 0.001). (**C**) Bray–Curtis-based PCoA analysis revealed significant differences in microbial community structure between the patients and their family members, with fewer individual differences in the NORA group. (**D**) The 34 highly expressed differential genera identified by the quasi-paired cohort method (Wilcoxon signed-rank test) for each subject. The color bar indicates the Z score-normalized abundance (scaled by row); green and red names represent low and high expression, respectively. (**E**) Relative abundance of 6 highly expressed differential genera. *Bifidobacterium* was found to be significantly depleted in RA patients. (**F**) List of highly expressed species and their correlation with clinical markers of systemic inflammation and titers of autoantibodies. (**G**) The correlation between Bifidobacterium and clinical parameters was computed by using Pearson’s correlation coefficient. (**H**) List of AKA-associated species and their correlation with clinical markers. (**I**) Abundance of *Bifidobacterium* in AKA (−) and AKA (+) subgroups. (**J**) Chao 1 index of clinical cohort II was significantly higher in RA patients compared to healthy controls. (**K**) Bray–Curtis-based PCoA analysis of clinical cohort II. (**L**) Abundance of Bifidobacterium in the 3 groups. Values are presented as means ± SEM. Unpaired t-tests were used for comparisons between the 2 groups. One-way ANOVA and Bonferroni’s multiple comparisons test were performed between multiple groups. The colored bar indicates Spearman’s coefficients: purple, *p* > 0, positive correlation; green, *p* < 0, negative correlation. The pair with a *p* < 0.05 was selected. Significant results are indicated as: * *p* < 0.05, ** *p* < 0.01.

To identify gut microbiota alterations due to the disease state of RA, we recruited a clinical exploratory cohort to analyze the species composition in fecal samples and define the differentially abundant genera by 16S rRNA sequencing and bioinformatics analysis (Figure 1A). The Chao 1 index revealed that the richness of gut microbes was markedly greater in RA patients than in their relatives (Figure 1B), and principal coordinates analysis (PCoA) based on Bray-Curtis heterogeneity highlighted clear differences in the structure of the microbial community between the two groups (Figure 1C). Among these microbes, 16 taxa, including *Bifidobacterium* and *Stenotrophomonas*, were found to be enriched in samples obtained from the RA patient family members (Figure 1D,E). Notably, *Bifidobacterium* abundance was negatively correlated with the levels of common clinical rheumatology laboratory and activity indicators, such as rheumatoid factors (RF), C-reactive protein (CRP), cyclic citrullinated peptide (CCP), clinical disease activity index (CDAI) and disease activity score 28 joint (DAS 28) (Figure 1F,G). In addition, the presence of anti-keratin antibody (AKA) has been shown to correlate with disease severity and activity, which is crucial for the early diagnosis and prognosis of RA [20]. Analysis of the correlation between the levels of abundant species and AKA levels revealed a greater richness of *Bifidobacterium* in the AKA-negative group than in the AKA-positive group, and there was a negative correlation between *Bifidobacterium* abundance and the levels of clinical indicators in the AKA-positive group (Figure 1H,I).

To further assess the variability of *Bifidobacterium* in RA and to explore its relevance to the severe sequelae of joint injury, we enrolled another clinical cohort (Figure 1A). As shown in Figure 1J,K, the Chao 1 index and PCoA analysis indicated marked differences in the richness and structure of the gut microbiota between the two groups in fecal samples. Next, we subgrouped the established patients again, based on the presence or absence of joint deformation, and compared the abundances of some species. *Bifidobacterium* abundance was significantly upregulated in NDJ patients (about 2.88 times higher than the DJ group) (Figure 1L). A high abundance of *Bifidobacterium* is strongly and negatively associated with disease activity and sequelae in RA patients and may serve as an effective target for the prevention and treatment of RA.

### 3.2. B. pseudocatenulatum Exhibits a Preventive Effect against Arthritis Progression in Mice

**Figure 2:**
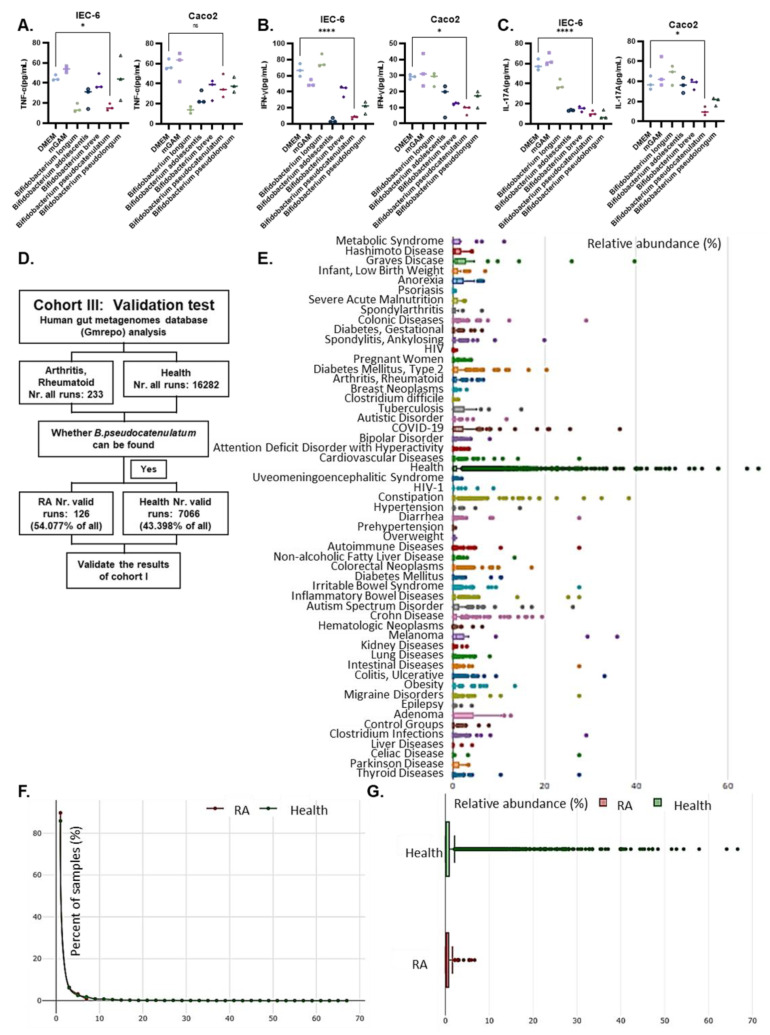
Co-culture experiments to identify specific Bifidobacterium strains and validate their function in the metagenomes database. (**A**–**C**) ELISA assay for the expression levels of TNF-α, IFN-γ, and IL-17A in culture supernatants of LPS-stimulated IEC-6 and Caco-2 cells were treated with 5 different *Bifidobacterium* strains. *B. pseudocatenulatum* significantly inhibited inflammatory cytokine secretion. Values are presented as means ± SD. A one-way ANOVA was used to compare values among multiple groups. Significant results are indicated as: * *p* < 0.05, **** *p* < 0.001, ns, non-significant. (**D**) Another validation cohort based on information from the metagenomes database of RA patients and healthy individuals. (**E**) Boxplot of the relative abundance of *B. pseudocatenulatum* in GMrepo, which was expressed in the healthy group above all disease groups. (**F**,**G**) Relative abundance of *B. pseudocatenulatum* in RA patients and healthy individuals verified in GMrepo.

To probe novel beneficial *Bifidobacterium* strains still more, five specific strains were selected for in vitro screening experiments, in which co-culture of *B. pseudocatenulatum* was found to have the strongest inhibitory effect on the secretion of inflammatory factors (such as IFN-γ and IL-17A) in IEC-6 and Caco-2 cells stimulated by lipopolysaccharide (LPS) (Figure 2A–C). Consistent with these findings, analysis of publicly available online data in the GMrepo database, a curated database of human gut metagenomes, showed that *B. pseudocatenulatum* was expressed in up to 54.08% of RA patients (43.398% of healthy individuals), with higher expression in the healthy group than in RA (Figure 2D–G). Therefore, our further studies focused on *B. pseudocatenulatum* as a promising probiotic that may play a beneficial role in inhibiting RA progression.

**Figure 3:**
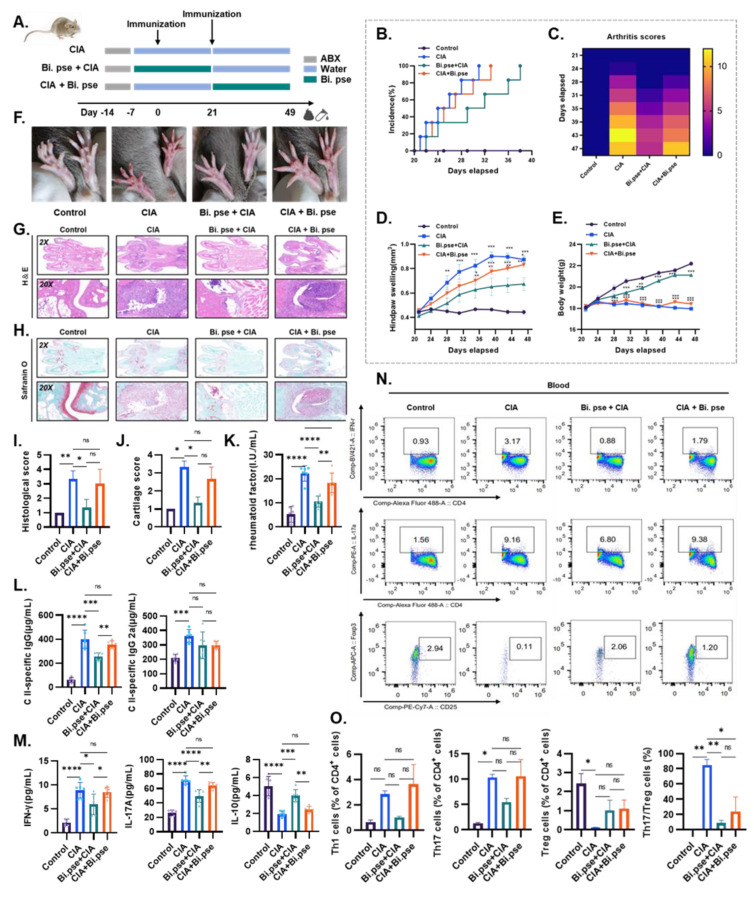
Effect of *B. pseudocatenulatum* on the onset and progression of arthritis in the CIA mouse model. (**A**) Flow chart showing the experimental treatment design. (**B**–**E**) Effects of *B. pseudocatenulatum* on CIA incidence, arthritis clinical scores, hind paw swelling, and body weight. Values are presented as means ± SEM. A two-way ANOVA was used to compare values among multiple groups. (**F**) Images of paws in each group before sacrifice. (**G**,**H**) H&E and Safranin O staining of mice paws to determine joint pathology and cartilage destruction (Scale bars set at 50 and 500 μm). (**I**,**J**) Evaluation of joint pathology and cartilage of mice in each group. Values are presented as means ± SD. A one-way ANOVA was used to compare values among multiple groups. (**K**) Levels of arthritis markers (RF) in mice serum were determined by ELISA. (**L**) The concentrations of anti-CII IgG and anti-CII IgG2a in mice serum were determined by ELISA. (**M**) The concentrations of pro-inflammatory factors IFN-γ and IL-17A, and the anti-inflammatory factors IL-10 in mice serum were determined by ELISA. (**N**,**O**) FCM analysis of the percentage of Th1 (CD4 + IFN-γ +), Th17 (CD4 + IL-17A +), Treg (CD4+CD25+foxp3+) cells, and ratio of Treg/Th17 in blood of mice. Values are presented as means ± SEM. One-way ANOVA and post-hoc Dunnett’s test were performed between multiple groups. Significant results are indicated as: * *p* < 0.05, ** *p* < 0.01, *** *p* < 0.001, **** *p* < 0.001, ns, non-significant.

Subsequently, we assessed in vivo in mice whether *B. pseudocatenulatum* had a preventive or therapeutic effect on the progression of RA (Figure 3A). Etanercept, a widely used DMARD, was used as a positive control. The incidence of arthritis reached 100% in the CIA model, prevention, and treatment groups on days 31, 33, and 38 after initial immunization, respectively, and early gavage of *B. pseudocatenulatum* noticeably delayed disease onset (Figure 3B). After booster immunization, the disease status of the mice was evaluated by scoring arthritis severity based on clinical quantitative scoring rules and assessments of swelling in the hind paws. The CIA model group showed erythema on day 20 postimmunization, with a continued increase in paw thickness and weight loss, and a rapid increase in clinical scores at a later stage (Figure 3C–E). Excitingly, disease onset was markedly slowed in the prevention group, and the degree of disease progression was milder and similar to that observed in the etanercept-treated group (Figure 3B, Supplementary Figure S1A,B). The occurrence of arthritis symptoms in the treatment group was analogous to that in the CIA group, with little or no amelioration.

The damage phenotype of the paws in each group of mice is shown in Figure 3F, while we used hematoxylin and eosin (H&E) and safranin O staining to assess the effect of *B. pseudocatenulatum* on joint pathology and cartilage destruction (Figure 3G,H). A substantial influx of inflammatory cells and considerable destruction of cartilage, proliferation of granulation tissue, lymphocyte infiltration, and chronic inflammation were observed in all CIA mice and the *B. pseudocatenulatum* treatment group, while the prevention group exhibited few of these symptoms and significantly lower histological and cartilage scores. As expected, animals in the prevention group also showed significantly lower histological and cartilage scores than the treatment group (Figure 3I,J). Taken together, these findings imply that early gavage of *B. pseudocatenulatum* is potent in delaying the incidence of disease and preventing joint destruction.

### 3.3. B. pseudocatenulatum Inhibited Specific Antibodies and Proinflammatory CD4+ T Cells

Next, we measured the concentrations of RF in mice serum by turbidimetric assay, and the results showed that the expression levels of this marker in the *B. pseudocatenulatum* prevention group were 0.5-fold lower than the treatment group (Figure 3K). Furthermore, the measurement of antibody concentrations showed that the levels of anti-CII immunoglobulin G (IgG) were significantly lower in the *B. pseudocatenulatum* prevention group than those in the CIA group, but the treatment group did not show significantly decreased IgG levels, and IgG2a levels did not differ dramatically between groups (Figure 3L). In addition, our analyses showed that the pro-inflammatory cytokines IL-17A and IFN-γ were significantly lower in the prevention group compared to the treatment group, while the anti-inflammatory cytokine IL-10 was higher (Figure 3M).

We proceeded to investigate the effect of *B. pseudocatenulatum* on the immune balance in CIA mice. After administration of *B. pseudocatenulatum* for 28 days, we measured the numbers of CD4+ T cells in the peripheral blood of mice. As shown in Figure 3N,O, compared to the normal mice, the number of cells in the Th1 (IFN-γ+ in CD4+ T cells) and Th17 (IL-17+ in CD4+ T cells) populations was significantly increased, while the number of Treg (CD25+Foxp3+ in CD4+ T cells) cells was significantly decreased in CIA mice. In contrast, the ratio of Th17/Treg cells of the CIA vehicle group was significantly higher than that in the normal group. However, compared with mice in the CIA vehicle group, the numbers of Th1 and Th17 cells in the *B. pseudocatenulatum* prevention group were reduced, the numbers of Treg cells were increased. Note that the ratio of Th17/Treg cells was significantly lower in both the *B. pseudocatenulatum* prevention group and treatment group compared to the CIA group. Thus, we conclude that *B. pseudocatenulatum* plays a more pronounced role in preventing joint damage while improving serological inflammatory symptomatology and immune balance in CIA mice.

### 3.4. B. pseudocatenulatum Protected the Intestinal Barrier and Increased BSH-Enriched Bacteria

**Figure 4:**
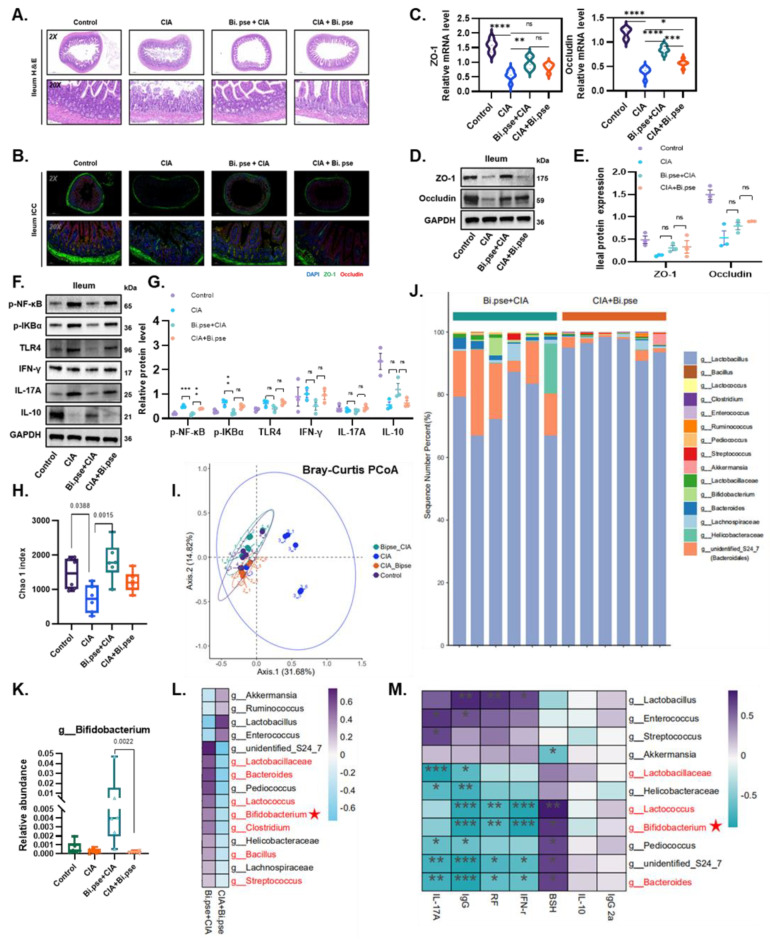
*B. pseudocatenulatum* protects intestinal barrier function and reshapes the gut microbiota. (**A**) Histopathological changes in ileum tissues with HE staining (scale bars set at 50 and 500 μm). (**B**) Immunofluorescence-stained sections of ileum using antibodies against ZO-1 (green) and Occludin (red) with DAPI (blue) as the counterstain (scale bars set at 50 and 500 μm). (**C**) Quantitative RT-PCR and (**D**,**E**) Western Blot showing expression of ZO-1 and Occludin. (**F**) Representative Western Blot image and (**G**) analysis of the alternative NF-κB signaling pathway. (**H**) Alpha-diversity analysis (Chao 1 index) of fecal microbiota OTU at various taxonomic ranks. (**I**) PCoA of β-diversity based on the Bray–Curtis dissimilarity matrix of OTU-level compositional profiles. (**J**) The gut microbial composition profiles at the genus level. (**K**) Abundance of *Bifidobacterium* in the 4 groups. (**L**) Heatmap of differentially abundant bacterial OTUs using one-way ANOVA, at *p* < 0.05. BSH-enriched genera are marked in red; green and purple names represent low and high expression, respectively. (**M**) Associations of the abundance of differential genera with serum cytokines. The colored bar indicates the Spearman’s coefficients: purple, *p* > 0, positive correlation; blue, *p* < 0, negative correlation. Red font indicates BSH-enriched genus. Values are presented as means ± SEM. One-way ANOVA and post-hoc Dunnett’s test were performed between multiple groups. Significant results are indicated as: ns: not significant, * *p* < 0.05, ** *p* < 0.01, *** *p* < 0.001, **** *p* < 0.001.

Clinically, intestinal barrier dysfunction is an initiator of RA arthropathy [21,22]; indeed, we observed some degree of ileal damage in CIA mice (Figure 4A). Surprisingly, in our study, the *B. pseudocatenulatum* intervention used in mice in both the prevention and treatment groups effectively protected the ileal barrier and significantly increased the expression levels of the tight junction (TJ) proteins ZO-1 and Occludin in ileal tissue (Figure 4B–E). Moreover, the prevention group exhibited further suppression of the expression of inflammatory factors through the inhibition of the NF-κB pathway, which is highly relevant to autoimmunity and has been reported to influence the inflammatory mediators of RA; this effect was not observed in the treatment group (Figure 4F,G). These findings further suggest that *B. pseudocatenulatum*, a single bacterium, could prophylactically slow the onset of arthritis and ameliorate the early stages of the disease by maintaining intestinal homeostasis. However, the elucidation of the exact mechanism behind these effects needs further experimentation.

We further characterized the effect of *B. pseudocatenulatum* on the overall structure and diversity of the intestinal flora by analyzing the microbial 16S rRNA gene sequences in the feces of mice in the prevention and treatment groups. The Chao 1 index was significantly higher in the *B. pseudocatenulatum* prevention group (Figure 4H). Bray-Curtis PCoA revealed significant clustering of the intestinal microbial communities in both experimental groups, and both were closer to the control than the CIA group (Figure 4I).

Several genus-level changes in relative abundance were also observed (Figure 4J). Operational taxonomic units (OTUs) of *Bifidobacterium* were remarkably higher in the prevention group than other groups, denoting a superior colonization rate of *B. pseudocatenulatum* in CIA mice subjected to early gavage (Figure 4K). In addition, OTUs of *Lactobacillaceae* spp., *Bacteroides* spp., *Lactococcus* spp., *Clostridium* spp., *Bacillus* spp., and *Streptococcus* were also observed to be significantly higher in the prevention group than in the treatment group (Figure 4L). It has been shown that the common function among all identified microbial genera is the enrichment of BSH which can mediate BSH enzyme activity in the gut [13]. These discoveries indicate the importance of further exploring whether the role of *B. pseudocatenulatum* in maintaining intestinal homeostasis is based on the synergistic involvement of these differential bacteria in regulating BSH activity as well as BAs pool. To further determine the relevant functions of these groups, we performed an analysis of the correlation between the differentially abundant genera and the levels of various molecules. We found that the levels of BSH-enriched genera, particularly *Bifidobacterium*, were negatively correlated with the levels of proinflammatory factors but positively correlated with the levels of BSH and anti-inflammatory factors (Figure 4M), suggesting that these genera possess cooperative anti-inflammatory potential.

### 3.5. B. pseudocatenulatum Prophylactic Intervention Enhanced BSH Activity and the Accumulation of Unconjugated Secondary BAs

**Figure 5:**
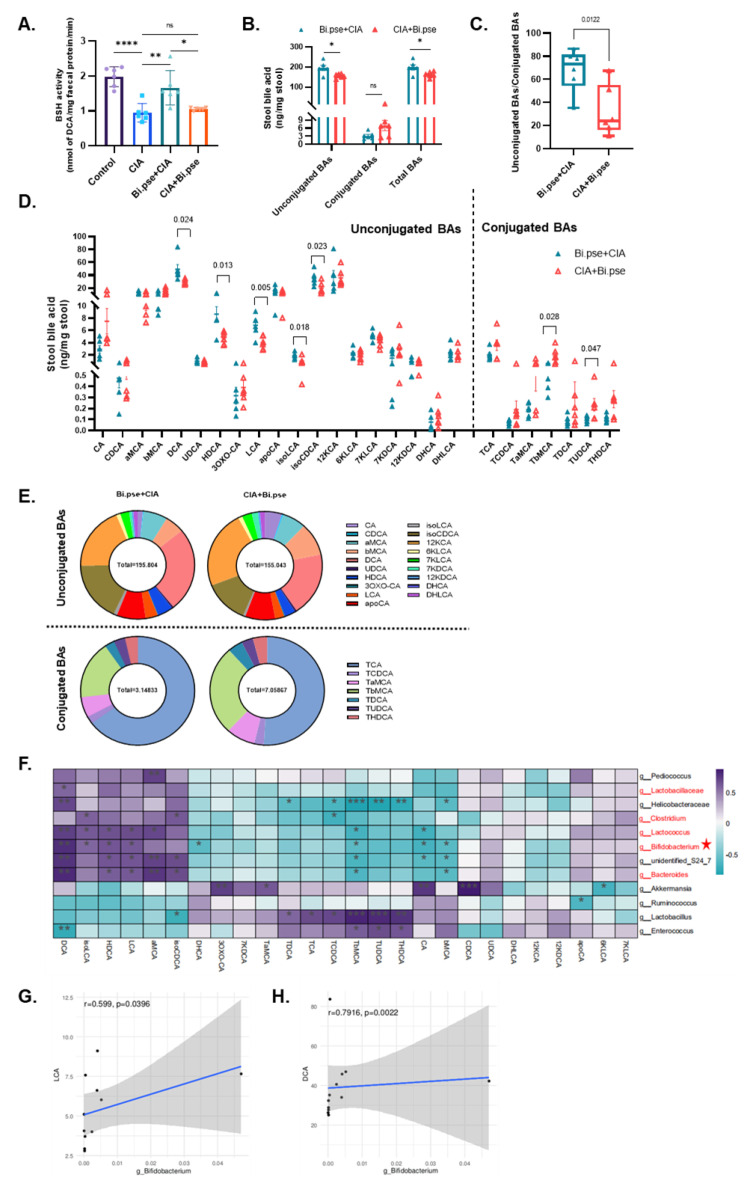
Upregulation of BSH activity and accumulation of unconjugated BAs in *B. pseudocatenulatum* prevention group. (**A**) A precipitation-based assay was established to measure fecal BSH activity. (**B**,**C**) Fecal unconjugated BAs, conjugated BAs, and the ratio of unconjugated to conjugated BAs compared between prevention and treatment groups. (**D**) BAs in feces were quantified using targeted metabolomics. (**E**) Fecal relative BA pool composition. (**F**) Heatmap showing positive (**purple**) and opposite (**blue**) correlation between BAs and the 12 genera. (**G**,**H**) *Bifidobacterium* spp. are strongly correlated with the expression levels of both DCA and LCA by using Spearman’s correlation coefficient. Red font indicates BSH-enriched genus. Values are presented as means ± SEM. One-way ANOVA and post-hoc Dunnett’s test were performed between multiple groups. Significant results are indicated as: ns: not significant, * *p* < 0.05, ** *p* < 0.01, *** *p* < 0.001, **** *p* < 0.001.

A precipitation-based assay was established to measure BSH activity in mouse feces. The results showed a significant enhancement of BSH activity in the prevention group (Figure 5A). A targeted metabolomics approach based on UPLC-MS/MS was established to characterize the metabolic profile of BAs in mouse feces. The analysis revealed that in the *B. pseudocatenulatum* prevention group, the proportion of unconjugated to conjugated BAs was 2.09-fold higher than that in the treatment group, and the acids with the most significantly elevated levels were DCA, LCA, hyodeoxycholic acid (HDCA), isoLCA and iso-chenodeoxycholic acid (isoCDCA), whereas the levels of taurine-bound BAs were generally lower and glycine-bound BAs were generally undetected (Figure 5B–E). Furthermore, a genus-level analysis of the correlation between microbial taxonomic composition and BAs suggested that the levels of the highly expressed deconjugated BAs indicated above were significantly and positively correlated with the levels of five genera with high expression of BSH enzymes, including *Bifidobacterium* spp., *Lactobacillaceae* spp., *Clostridium* spp., *Lactococcus* spp., and *Bacteroides* spp. (Figure 5F). In particular, the abundance of *Bifidobacterium* was strongly correlated with the levels of both LCA and DCA (Figure 5G,H). This finding suggests that the protection of the intestinal barrier and inhibition of intestinal inflammation in the ileum by the early gavage of *B. pseudocatenulatum* might be attributed to increased concentrations of deconjugated bile acids, such as LCA and DCA.

### 3.6. B. pseudocatenulatum-Promoted DCA Inhibits Alternative NF-κB Pathway by TGR5-Regulated Inflammation

**Figure 6:**
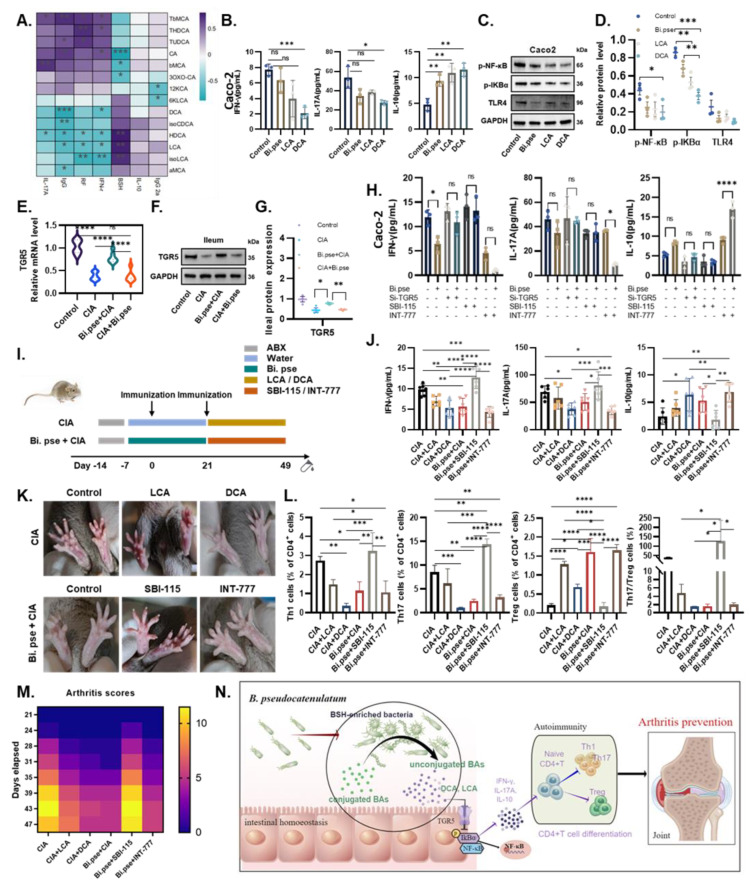
*B. pseudocatenulatum*-promoted DCA inhibits the alternative NF-κB pathway by regulating TGR5 receptor expression. (**A**) Heatmap of positive (purple) and opposite (blue) correlation between BAs and cytokines. (**B**) The expression levels of IFN-γ, IL-17A, and IL-10 in culture supernatants of LPS-primed Caco-2 cells treated with *B. pseudocatenulatum*, LCA, and DCA. (**C**,**D**) Western blot shows the expression of NF-κB signaling pathway molecules. (**E**) Quantitative RT-PCR and (**F**,**G**) Western blot showing expression of ileum TGR5. (**H**) INT-777, a semi-synthetic TGR5 agonist, greatly enhanced the effect of *B. pseudocatenulatum* in inhibiting IL-17A and promoting IL-10 in LPS-stimulated Caco-2 cells. SBI-115, a TGR5 inhibitor, and si-TGR5 offset the inflammatory homeostatic effect of *B. pseudocatenulatum*. (**I**) Flow chart showing another experimental treatment design. (**J**) The concentrations of pro-inflammatory factors IFN-γ and IL-17A, and the anti-inflammatory factors IL-10 in mice serum were determined by ELISA. (**K**) Representative images of paws in each group before sacrifice. (**L**) FCM analysis of the percentage of Th1 (CD4 + IFN-γ +), Th17 (CD4 + IL-17A+), Treg (CD4+CD25+foxp3+) cells, and ratio of Treg/Th17 in blood of mice. (**M**) Arthritis clinical scores of CIA mice. (**N**) Graphical abstract of this study. Based on the interactions of the gut–joint axis, *B. pseudocatenulatum* increased the levels of unconjugated secondary bile acids and protected the intestinal barrier to activate the TGR5-mediated immune response, preventing RA symptoms. Values are presented as means ± SD. One-way ANOVA and post-hoc Dunnett’s test were performed between multiple groups. Significant results are indicated as: ns: not significant, * *p* < 0.05, ** *p* < 0.01, *** *p* < 0.001, **** *p* < 0.001.

Finally, to clarify the role of BAs in the regulation of inflammation, we investigated the correlation between cytokine and BA levels. The analysis showed that LCA and DCA levels were significantly negatively correlated with the levels of proinflammatory factors, such as TNF-α and IL-17A, and positively correlated with the levels of the anti-inflammatory factor IL-10 (Figure 6A). We subsequently experimentally demonstrated that DCA dramatically reduced the expression of inflammatory factors in Caco-2 cells and inhibited critical inflammation-generating receptors and pathways, namely the TLR4/NF-κB pathway, while the effect of LCA was unremarkable (Figure 6B–D).

We next examined the mRNA and protein expression levels of TGR5 in mouse ileal tissue and found that TGR5 expression was significantly increased in the *B. pseudocatenulatum* prevention group (Figure 6E–G). Similarly, the use of INT-777, a semi-synthetic TGR5 agonist, greatly enhanced the effect of *B. pseudocatenulatum* in inhibiting IL-17A expression and promoting IL-10 expression in LPS-stimulated Caco-2 cells (Figure 6H). In contrast, the inflammatory homeostatic effect of *B. pseudocatenulatum* was lost in the presence of the TGR5 inhibitor SBI-115 or TGR5-targeting siRNAs in vitro (Figure 6H). More conclusively, we administered INT-777 and SBI-115 after early gavage of *B. pseudocatenulatum* in CIA mice and found that the tendency of TGR5 agonists and inhibitors to modulate inflammatory factors in in vitro experiments could be verified to be repeatably reproduced in animal experiments (Figure 6I,J). Furthermore, DCA and INT-777 prominently suppressed the extent of paw injury, disease course, as well as Th1 and Th17 cell populations in the peripheral blood of mice, while promoting Treg cell populations (Figure 6K–M). In summary, it was revealed that after an early gavage of *B. pseudocatenulatum*, TGR5 receptors in CIA mice were activated mainly by increases in the DCA levels and the inhibition of inflammatory pathways and proinflammatory T cells to prevent the worsening of joint injury symptoms.

## 4. Discussion

Numerous studies have shown that the use of intensive immunosuppressive-based treatment strategies during the early stages of RA contributes to prolonged remission and favorable function, imaging, and prognostic outcomes. The clinical “window of opportunity” concept has been proposed to highlight the significance of early intervention in patients with RA [23], but strategies that focus on susceptibility factors are limited. In this study, we investigated the use of a single probiotic, *B. pseudocatenulatum*, which can be used as a prophylactic treatment for arthritis. Due to its apparent efficiency and low toxicity, *B. pseudocatenulatum* supplementation may prove to be an efficient candidate to suppress the onset of RA at a very early stage and improve immunosuppressive efficacy (Figure 6N). Moreover, it must be emphasized that the timing of intervention is an important factor that affects the efficacy of using probiotics as prophylactics, and that any treatment used to control the gut microbiota should perhaps be administered during or before the onset of the disease.

Previous studies have demonstrated that severe gut microbial dysbiosis occurs in RA patients; however, the changes in the gut microbiota that occur during the initiation, development and management of RA remain to be elucidated. In the current study, we revealed for the first time that pretreatment using a specific gut microbial strain, *B. pseudocatenulatum*, restores the disorganized microbiome due to arthritis at the genome-wide level. The salutary role of *B. pseudocatenulatum*, as demonstrated in the prevention group, may be attributed to its contribution to promoting synergistic activity among BSH-enriched genera. These genera synergistically repaired the intestinal barrier and regulated BA metabolic pathways in the prevention group, suggesting that *B. pseudocatenulatum* is a pioneer species that enables the colonization of other probiotic species to promote a favorable intestinal ecosystem. Furthermore, we found that this probiotic relay from *Bifidobacterium* to *Lactobacillus* was sensitive to the intestinal inflammatory state determined by CD4+ T cells. Overall, we found that the introduction of *B. pseudocatenulatum* into the mouse models profoundly altered the microbiome, suggesting a dynamic and interconnected ecosystem.

Probiotics play an integral role in promoting bone health by facilitating the production of useful metabolites, such as BAs [24]. In this study, the prevention group showed a significant increase in the concentration of total BAs, with the highest absolute levels of deconjugated BAs (LCA and DCA). Notably, only deconjugated BAs, but not conjugated BAs (CA and CDCA), can inhibit the secretion of proinflammatory cytokines and are key regulators in the development of autoimmune diseases [25]. The anti-inflammatory effects of deconjugated BAs have been observed in chronic cholestasis, and they have been shown to reduce the production of proinflammatory factors, such as IL-1β, IL-6, and TNF-α [26]. These effects are initiated through the activation of BA receptors, including G protein-coupled receptors (GPCRs) and nuclear receptors [27]. More specifically, deconjugated BAs are the preferred ligands for TGR5, a specific GPCR [28]. We found that TGR5 expression was increased in the prevention group. A previous study in macrophages reported that the inhibitory inflammatory effect of BAs via TGR5 was mediated through the NF-κB pathway [29]. Moreover, TGR5 signaling inhibited NF-κB activation in macrophages via the cAMP-PKA pathway, and this inhibition was dependent on CREB-induced IL-10 secretion [22]. In our study, we also observed that the *B. pseudocatenulatum* prevention group exhibited inhibited activation of NF-κB via the TGR5 receptor, thus promoting the expression of the inflammatory factor IL-10 and inhibiting the secretion of proinflammatory factors; however, a clear causal relationship needs to be confirmed through further experimentation.

Several therapeutic regimens, such as the use of salazosulfapyridine and minocycline [22], have been incorporated into DMARD regimens for treating RA, suggesting that an optimal microbiota can effectively optimize the immune response [30]. In addition, probiotics have been shown to promote the efficacy of immunosuppressive agents such as anti-PD-L1 therapy, an immune checkpoint blocking approach [31]. In one study, it was suggested that probiotics such as *Lactobacillus casei* may have the potential to help alleviate RA symptoms and inhibit the production of proinflammatory factors in patients treated with DMARDs [32]; this implies the existence of a synergistic effect of DMARDs and probiotics in the treatment of RA, an aspect that we did not explore in the present study. Therefore, we anticipate future studies focusing on the potential role of probiotics in RA, especially in combination with investigations of the human genome, and their contribution to early disease diagnosis and in improving the efficacy of immunosuppressive agents.

Overall, this study suggests that the introduction of *B. pseudocatenulatum* before the onset of arthritis may rebalance the immune response and improve systemic inflammation and autoimmunity by modulating gut-related characteristics, such as the gut microbiota composition, BA metabolism, and the intestinal barrier, thereby inhibiting joint damage. We predict that extensive research will emphasize the prospective effects of intestinal probiotics; in particular, the use of individual bacteria for the early prevention and therapy of RA.

## 5. Conclusions

The current work demonstrates the innovative implication that the use of a single bacterium, *B. pseudocatenulatum*, can prevent the pathogenesis of RA by modulating intestinal homeostasis and immune metabolism (Figure 6N). Our findings support the prevailing idea that probiotics may be a new strategy for preventing and treating RA in humans. Notably, these results were obtained in an experimental mouse model of arthritis; hence, in-depth studies of RA patients are greatly warranted to verify our findings and to assess whether this approach would dramatically improve the prognosis of patients. Finally, the precise molecular mechanisms underlying the preventive and therapeutic effects of probiotics and the new bacterial markers identified in the gut deserve further experimentation, which will facilitate the use of microbiology combined with genetics for precision medicine in RA.

## Data Availability

The original contributions presented in the study are included in the article/Supplementary Material, further inquiries can be directed to the corresponding authors.

## Supplementary Materials

The following supporting information can be downloaded at: https://www.mdpi.com/article/10.3390/nu15020255/s1. Figure S1: The incidence of arthritis and clinical scores in all groups; Table S1: Information on Cohort I: Discovery test—A clinical exploratory study; Table S2: Information on Cohort II: Validation test—Another clinical retrospective study.
