# Supplementary material for: Bifidobacterium pseudocatenulatum-Mediated Bile Acid Metabolism to Prevent Rheumatoid Arthritis via the Gut–Joint Axis
Source: Nutrients. 2023 Jan 4;15(2):255. doi: 10.3390/nu15020255 (PMC9861548; doi:10.3390/nu15020255)
Supplement: Supplementary file 1 [file nutrients-15-00255-s001.zip › nutrients-2052981-supplementary.pdf]

Supplementary material

Material and methods

1.1. Human cohorts

This study was performed in according to established ethical guidelines and was approved by the Research Ethics Committee of West China Hospital, Sichuan University (Chengdu, China). All subjects obtained a written informed consent prior to the start of the study.

Clinical cohort I recruited stool samples from 36 treatment-naïve new-onset RA patients (NORA) and 11 healthy family members, and the patient group was further divided into positive and negative groups based on the presence of anti-keratin antibody (AKA). Clinical characteristics were shown in Table S1.

Clinical cohort II recruited 40 well-established RA patients with disease-modifying antirheumatic drugs (DMARDs) treatment and 34 healthy individuals, which were further divided into DJ (deformed joints; n = 18) and NDJ (no deformed joint; 3 years after diagnosis, n = 22) group based on the presence or absence of sequelae of joint damage after drug administration. Clinical characteristics were shown in Table S2. All samples were stored at −80 °C until further analyses.

Clinical cohort III analyzed 233 records of RA and 16282 records of Healthy in GMrepo database（a curated database of human gut metagenomes), and 126 records of RA and 7066 records of Healthy were filtered for further analysis based on whether expressed *B. pseudocatenulatum*.

1.2. Bacterial strain and culture condition

Bifidobacterium pseudocatenulatum (CGMCC 1.2277), *Bifidobacterium longum* (CCFM 1029), *Bifidobacterium adolescentis* (CGMCC 1.2190), *Bifidobacterium breve* (ATCC 15700), *Bifidobacterium pseudolongum* (BNCC 135158) was cultured anaerobically (10% CO2, 10% H2 and 80% N2) in modified Gifu Anaerobic Medium (mGAM, Nissui Pharmaceutical Co., Ltd., Tokyo, Japan) at 37 ºC for 24–36 h.

Prior to use in animal experiments, the bacterial cultures were centrifuged at 4000g for 15 min and washed twice with sterile saline solution. The bacteria were then re-centrifuged and re-suspended in a 30% (v/v) sucrose solution. The bacterial suspension was stored at −80 °C, and the viability was measured by colony counting prior to use. A fresh *B. pseudocatenulatum* suspension was prepared daily by diluting to a suspension with 1×10^{9} living cells before the experiments.

1.3. Co-Culture model

Intestinal epithelial IEC-6 cells and Caco-2 cells were purchased from ATCC (Ma-nassas, VA, USA) and cultured at 37 °C, 5% CO2 in DMEM (Gibco, Grand Island, NY, USA) with 10% heat-inactivated fetal bovine serum (FBS, Gibco, Grand Island, NY, USA). Caco-2 cells are human clonal colon adenocarcinoma cells, structurally and functionally similar to differentiated small intestinal epithelial cells.

After apposition, cells were incubated for 6 h under lipopolysaccharide (LPS, 1 μg/mL, Sigma-Aldrich, St. Louis, MO, USA) stimulation to produce pro-inflammatory cytokines. Subsequently, a final concentration of 106 CFU/mL of live bacterial suspen-sion was added to

the medium. Culture samples were collected after 6–8 h to detect the secretion of TNF-α, IFN-γ, and IL-17A.

1.4. Animal experiments

Six-week-old male DBA/1J mice were purchased from Changzhou Cavens Exper-imental Animal Co., Ltd. (Jiangsu, China). All of the experiment processes and animal cares were carried out in accordance with the regulations of the Animal Ethics Committee of Xiangya Hospital, Central South University (Hunan, China). After adaptation, all mice were given antibiotics treatment (ABX) for 7 days to deplete the gut microbiota using drinking water containing 1 g/L neomycin, 1 g/L ampicillin, 1 g/L metronidazole, and 0.5 g/L vancomycin. Then, the different experimental groups were established.

One schedule of the experiment is shown in Figure 3A. Daily oral administration with 0.2 mL *B. pseudocatenulatum* cocktail (OD600 of 1.0, corresponding to 1×10^{9} CFU) was done to the prevention group (Bi.pse+CIA, n=6) for 21 days starting on day 7 before the first immunization. The treatment group (CIA+Bi.pse, n=6) was given *B. pseudocatenulatum* cocktail after the booster immunization for 21 days through daily oral gavage. Meanwhile, controls and CIA mice were treated with saline through oral gavage during the whole trial.

Another schedule of the experiment is shown in Figure 6I. Daily oral administration with 0.1 mL *B. pseudocatenulatum* cocktail for 21 days starting on day 7 before the first immunization. After the booster immunization, deoxycholic acid (DCA, 50 mg/kg/day), lithocholic acid (LCA, 50 mg/kg/day), INT-777 (a semi-synthetic TGR5 agonist, 80 mg/kg/day), and SBI-115 (a TGR5 antagonist, 80 mg/kg/day, MedChemExpress, Monmouth Junction, NJ, USA) was given for 28 days, respectively.

On day 50 of the experiment, all mice were deeply anesthetized with 4% isoflurane (Abbott, Cham, Switzerland) by inhalation, whole blood was collected in test tubes by removing eyeball, and mice were sacrificed quickly by cervical dislocation, and ileal tissue and paw samples were collected from each mouse.

1.5. Induction and assessment of collagen-induced arthritis (CIA) mouse model

The CIA mouse model was created by administering DBA/1J mice with bovine type II collagen (2 mg/ml, Chondrex, USA) emulsified in complete Freund's adjuvant (CFA, 4 mg/ml, Chondrex, USA). This was followed by boosting with type II collagen emulsified in incomplete Freund's adjuvant (IFA, 4 mg/ml, Chondrex, USA) 21 days after the initial treatment.

Treated mice were monitored every 3 days for signs of arthritis based on paw swelling, body weight, and arthritis scores. Arthritis was scored on a scale of 0 to 4 based on the occurrence of swelling or inflammation on each of the paws using the scale based on previous reports. Arthritis was scored on a scale of 0 to 4 based on the occurrence of swelling or inflammation on each of the paws using the following scale: 0, normal, 1, perceptible swelling in a single digit, 2, swelling in more than one digit, 3, swelling of all digits and instep, and 4, severe swelling of the paw and ankle. The arthritis score for each mouse was expressed as the sum of the scores of all four paws, up to a maximum of 16 points. Hindpaw swelling was measured with vernier calipers.

1.6. Histological assessment of paws

The paws were fixed in 4% paraformaldehyde solution and were then decalcified using 10% EDTA for one month. After that, the paws were paraffin-embedded, and tissue sections were prepared and stained with hematoxylin-eosin (H&E) and Safranin O. The paws were grading

the severity of arthritis from 0 to 4 based on the intensity of lining layer hyperplasia, monocyte infiltration and pannus formation.

1.7. Serological cytokine and antibodies assay

The serum concentrations of pro-inflammatory factors (TNF-α, IFN-γ, and IL-17A) (Jianglai, Shanghai, China), and anti-CII antibodies (IgG and IgG2a) (Chondrex Inc., Redmond, WA, USA) were measured using Enzyme-Linked Immunosorbent Assay (ELISA) kits. In brief, samples, bio-antibody and streptavidin-HRP were added into 96-well plates pre-coated with antibody and incubated at 37 °C, respectively. After 60 min, liquid was discarded and washed completely. Next, chromogen solution was poured into to each well and preservation for 15 min at 37 °C to evade the light. Finally, stop solution was infused to each well and absorbance was measured at 450 nm.

Serum rheumatoid factor (RF) were measured by turbidimetric method following the instructions provided in the commercial kits (Coral clinical systems-Tulip diagnostics, Goa, India). Values >20 IU/ml were considered positive for RF. Each sample was assayed in duplicate.

1.8. Flow cytometry

Cells from blood samples were characterized based on cell surface markers using fluorescence-activated cell sorting (FACS) analyses. The cells were stained with dif-ferent fluorescently labeled monoclonal antibodies (mAb, Biolegend). In brief, 5×10^5 cells suspended in 100 μL of PBS with brefeldin A (Biolegend 423303) were mixed with 10 μL of 1640 medium and were incubated in the dark at 37 °C for 5 hours. The cell pellets were washed twice with PBS containing 2% BSA and were resuspended in PBS. Subsequent flow cytometry analysis was done immediately using the mouse an-ti-human IL-17A-PE mAb (Biolegend 506904), IFN-γ-BV421 mAb (Biolegend 505830), CD3-APC mAb (Biolegend 155606), CD8-PECY7 mAb (Biolegend 980910), CD4-FITC mAb (Biolegend 100529), CD25-PECY7 mAb (Biolegend 102016), FOXP3-AF647 mAb (Biolegend 320214), and CD45-APC-CY7 mAb (Biolegend 103154), Live-dead (Biolegend 423101).

The fluorescence intensity of the cells was evaluated by EPICS-XL flow cytometer (BD Biosciences, Franklin Lakes, NJ, USA). FlowJo V10 was used to further analyze the levels of Th1, Th17 and Treg cells. The following markers were used to identify different immune cell subsets: CD4+IFN-γ+ for Th1, CD4+IL-17A+ for Th17 cells, CD4+CD25+foxp3+ for Treg.

1.9. Quantitative Real-Time PCR

Total RNA was extracted from ileum tissues by using the TRIzol Reagent (Invitrogen, CA, USA). The PrimeScript RT reagent Kit (Takara, Dalian, China) was used to generate cDNA. Quantitative real-time PCR was performed with the QuantStudio 5 Real-Time PCR system (Thermo Fisher Scientific, USA) by using the iTaq Universal SYBR Green Supermix (BIO-RAD, CA, USA). Relative mRNA expression was calculated with the $2^{-\triangle\triangle}$ct method. The house-keeping gene GAPDH was used as reference.

The sequences of primers used are the following: ZO-1 forward, 5′-GCCGCTAAGAGCACAGCAA-3′ and reverse, 5′- GCCCTCCTTTTAACACATCAGA-3′; Occludin forward, 5′- TGAAAGTCCACCTCCTTACAGA-3′ and reverse, 5′-CCGGATAAAAAGAGTACGCTGG-3′; TGR5 forward, 5′- CCTGGCAAGCCTCATCGTC-3′ and reverse, 5′- AGCAGCCCGGCTAGTAGTAG-3′; GAPDH forward, 5′-AGGTCGGTGTGAACGGATTTG-3′ and reverse, 5′- GGGGTCGTTGATGGCAACA-3′.

1.10. Western blotting

Ileal tissue or Caco2 cell samples were lysed with IP lysis solution (Beyotime, Shanghai, China) containing PMSF (1 mM, Beyotime, Shanghai, China) for 5 min on ice, followed by centrifugation at 14,000 g, 5 min. Protein concentrations were quantified using the BCA Protein Assay Kit (Pierce, Rockford, IL, USA). 10 μg/μL of protein extracts were were prepared in a system with loading buffer (Beyotime Technology, Shanghai, China) and denatured by heating in a metal bath at 100 °C for 5 min. Denatured proteins were resolved by 4-20% SDS-PAGE and shifted to PVDF Membranes (Millipore Corporation, Tullagreen, IRL). The membranes were blocked with Fast Blocking Solution for half an hour (Beyotime, Shanghai, China), incubated with primary antibodies at 4 °C overnight, and then incubation with secondary antibodies. The bands were visualized using Superkine ECL Chemiluminescent Substrate (Abbkine Scientific Co., Ltd, USA) on Bio-Rad Chemi-Doc (Bio-Rad, Hercules, CA). The gray-values of the bands were calculated using Image lab software and were normalized to GAPDH (1:10000, Abcam). The antibodies used for mouse tissue and the antibody dilutions were as follows: IFN-γ (1:1000, Abcam), IL-17A (1:1000, Affinity), IL-10 (1:1000, Abcam), TGR5 (1:1000, Abcam), p- NF-κB (1:2000, Proteintech), p-IKBα (1:1000, ZEN BIO), TLR4 (1:1000, Affinity), ZO-1 (1:1000, Abcam), Occludin (1:1000, Affinity).

1.11. Immunofluorescence of ileum

Ileal fragments of 5 mm × 5 mm were frozen in liquid nitrogen, and 10 μm frozen sections were cut and fixed with cold acetone at 4 °C for 10 min. After three extensively washed with pre-chilled PBS, the sections were blocked with 10% serum in PBS and then incubated with the antibodies against ZO-1 (1:1000, Abcam) and Occludin (1:1000, Affinity) overnight at 4 °C, followed by staining with FITC-labeled secondary antibodies. Stained frozen sections were examined with a laser confocal microscope, identifying ZO-1 (green) and Occludin (red) with DAPI (blue) as the counterstain.

1.12. 16S rRNA gene sequencing

Genomic DNA was isolated from mice fecal samples using the DNA Isolation Kit (QIAGEN, Hilden, Germany) and sequenced on the illumina novaseq platform. The 16S universal eubacterial primers 515F: GTGCCAGCMGCCGCGGTAA and 806R: GGACTACHVGGGTWTCTAAT were used to evaluate the microbial ecology of each sample on an Illumina MiSeq (Illumina, San Diego, CA) sequencing system (Bionovogene Co., Ltd., Suzhou, China). Microbiome bioinformatics were mainly performed with QIIME 2 2019.4 while the OTU clustering procedure following the Vsearch (v2.13.4) pipeline described here (https://github.com/torognes/vsearch/wiki/VSEARCH-pipeline). Briefly, raw sequence data were demultiplexed using the demux plugin followed by primers cutting with cutadapt plugin. Sequences were then merged，filtered and dereplicated using functions of fastq_mergepairs, fastq_filter, and derep_fulllength in Vsearch. All the unique sequences were then clustered at 98% (via cluster_size) followed by chimera removing (via uchime_denovo). At last, the non-chimera sequences were re-clustered at 97% to generate OTU representive sequences and OTU table. Representive sequences were aligned with mafft and used to construct a phylogeny with fasttree2. Alpha-diversity metrics (Chao1 (Chao, 1984), Shannon (Shannon, 1948a, b), Simpson (Simpson, 1949), beta diversity metrics (Bray-Curtis dissimilarity) were estimated using the diversity plugin. Taxonomy was assigned to ASVs using the classify-sklearn nai¨ve Bayes taxonomy classifier in feature-classifier plugin against the Silva v132 99% OTUs reference sequences.

1.13. Bile salt hydrolase (BSH) enzyme activity assay

The principle of the BSH assay is an adaptation of the precipitation-based assay. Taurodeoxycholic acid (TUDCA) was used as a substrate for the enzyme assay, and the results are therefore expressed as the rate of deoxycholic acid (DCA) formation. Stool samples (50 mg)

were dissolved in 250 μL of PBS and total stool protein was quantified using the BCA Protein Assay Kit (Pierce, Rockford, IL, USA). The assay was performed in 96-well culture plates incubated at 37°C, pH 5.8 for up to 8 hours. In a total volume of 200 μL, 500 μg of fecal protein was incubated with sodium phosphate buffer (pH 5.8, final concentration 0.02 mM) and taurodeoxycholic acid (TUDCA, final concentration 1 mM; Merck, Damstadt, Germany). To prevent evaporation during incubation, the wells were covered with 50 μL of light paraffin oil (0.85 g/mL; PanReacAppliChem, Barcelona, Spain). Samples were taken in triplicates and the precipitation of insoluble deoxycholic acid (DCA) was monitored by absorbance measurements at 600 nm (A600) using a microplate reader (MultiSkan Go, Thermo Scientific, Dartford, UK). Fecal proteins incubated with PBS were used as negative controls and fecal proteins incubated with different concentrations of DCA (Merck, Damstadt, Germany) were used to establish standard curves to quantify precipitate formation.

1.14. Quantification of bile acids in feces

20 mg mice stool samples were weighed, added to 1 mL water-methanol (5:5 v/v), vortex mixed, sonicated for 30 s and centrifuged, and 100 μL supernatants were collected and dried under a gentle stream of nitrogen. The dried metabolite residues were dis-solved in 200 μL of 50% methonal. 10 μL residues were injected for quantification by UPLC-MS/MS (Ab SCIEX, USA, Triple Quad 6500+). A ACQUITY UPLC HSST3 C18 analytical column (2.1 mm * 100 mm, 1.8 mm) was used for chromatographic separation with the mobile phases being a mixture of 0.1% formic acid and 5 mM ammonium ace-tate in water (A) and methanol (B). The column temperature was 50 °C and the flow rate was 0.2 ml min $^{-1}$. The gradient was set as follows: 58%-60% B at 0–10 min, 60–66% B at 10-15 min, 66–70% B at 15-20 min, 70-80% B at 20-25 min, 80%-90% B at 25-30 min, 90% B at 30-32 min, 90%-50% B at 32-36 min, and 50% B at 36-40 min. Thirty-eight BAs were involved in the quantification by UPLC-MS/MS. Concentrations of the detected BAs were calculated with internal standard calibration from the linearly regressed standard calibration curves of individual BAs. The lower limits of quantification were 0.08 nmol/mg for all the BAs.

1.15. Correlation analysis

Correlation analysis was conducted through the Cloud-Seq Biotech platform (Cloud-Seq Biotech Ltd., Shanghai, China), including the determination of the correlation coefficients between the relative abundance of arthritis-correlated cytokines and the bile acid levels, as well as the correlation coefficients between the cytokines and the relative abundance of identified microbes. The Pearson correlation coefficient and Spearman rank correlation coefficient were used for linear correlation analysis.

1.16. Statistical analysis

The data were analyzed using the Graphpad Prism 9.0 software (Graphpad Soft-ware Inc., San Diego, CA, USA). Data are shown as means ± SEM or mean ± SD, and the *P* values were calculated by Unpaired t-tests, one-way analysis of variance (ANOVA) and post-hoc Dunnett's test. Corrected P values were used to account for multiple testing. Statistical significance is indicated by asterisks (*): *$P < 0.05$, **$P < 0.01$, ***$P < 0.001$, ****$P < 0.0001$, ns, non-significant. All reported analyses were considered significant at P values < 0.05.

A.

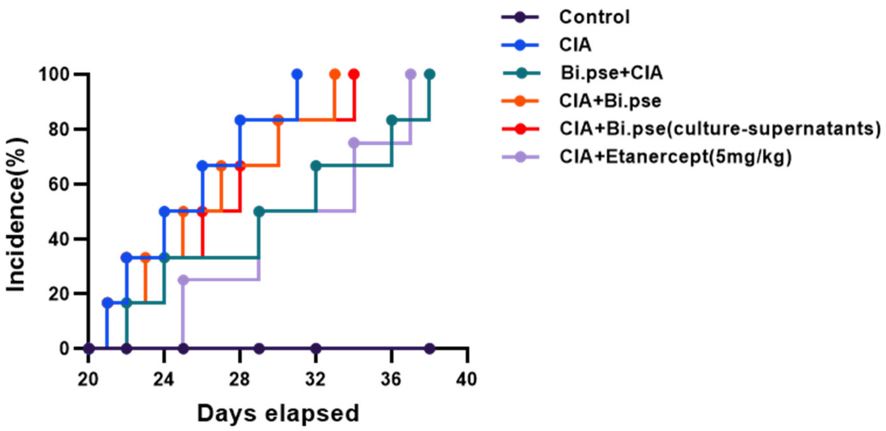

B.

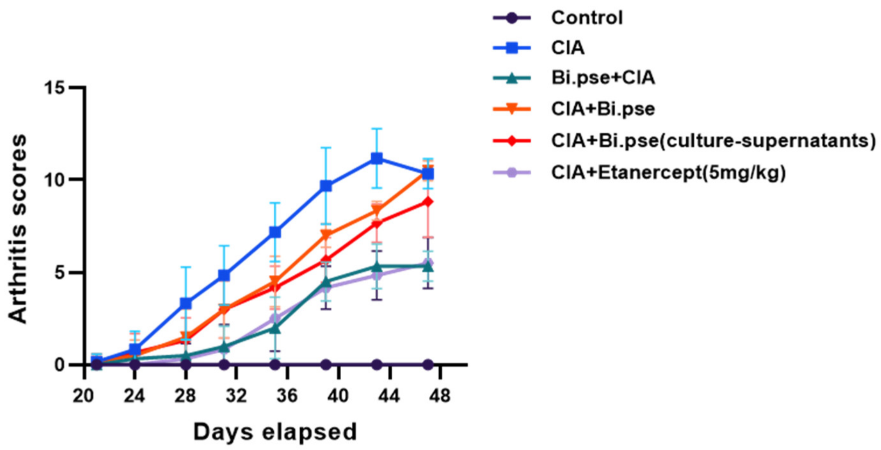

Figure S1. (A) The incidence of arthritis and (B) clinical scores in all groups. Etanercept, a widely used DMARD, was used as a comparative control.

Table S1. Information of Cohort I: Discovery test (A clinical exploratory study).

ID	Name	Family	CDAI	DAS28	AKA	RF	CRP	CCP
5	xx Liu	No	NA	NA	+	1820	NA	294.1
112	xx Nie	No	15	3.54	-	24.3	6.49	28.03
126	xx Zhu	Yes	17	4.26	+	190	15.4	>500.00
135	xx Gong	No	24	4.71	+	853	7.8	>500.00
138	xx ZHENG	No	NA	NA	-	718	1.44	NA
141	xx Wang	No	39	6.16	+	NA	36.9	349.3
154	xx Song	No	42	6.07	+	71.8	19	>500.00
175	xx Yan	No	42	6.66	-	82	48.4	168.2
187	xx Zhong	No	57	7.4	+	1920	60.4	330.6
188	xx Li	No	NA	NA	+	2020	142	424.5
190	xx Sha	No	38	5.76	+	245	14.2	443.6
198	xx He	Yes	45	5.77	-	166	77.2	362.7
199	xx Luo	No	20	4.63	-	<20.00	12.1	NA
202	xx Xiao	No	42	5.56	+	NA	4.22	152.7
204	xx Liu	No	28	4.49	-	98.7	13.3	265.4
205	xx Guo	No	19	3.87	-	285	16.6	30.23
215	xx Huang	No	13	3.2	-	<20.00	31.8	153.8
217	xx Yang	No	40	6.09	+	718	26.7	410.1
218	xx Zhou	No	37	6.32	-	NA	NA	328.1
221	xx Wang	No	41	6.21	+	205	16.3	>500.00
225	xx Li	Yes	45	5.55	+	71.8	10.4	>500.00
226	xx Chen	No	47	6.61	-	1760	84.3	41.02
227	xx Zhu	No	24	4.36	+	309	NA	>500.00
229	xx Nie	No	41	5.78	-	621	14.9	>500.00
238	xx Luo	No	22	NA	-	138	3.64	397.2
239	xx Yang	No	62	7.29	+	461	74.2	327.5
240	xx Ni	No	37	5.83	-	1170	25.5	>500.00
243	xx Chen	No	NA	NA	-	NA	NA	NA
250	xx Tang	No	40	6.46	-	NA	28.2	NA
251	xx Li	No	NA	7.47	-	620	56.2	>500.00
253	xx Xu	No	37	6.07	-	376	30.3	>500.00
258	xx Hu	Yes	45	6.32	+	100	NA	>500.00
259	xx Tan	Yes	27	5.55	-	460	9	NA
261	xx Zhang	Yes	73	7.5	+	101	14.4	>500.00
262	xx Zhao	Yes	57	7.2	-	NA	5.88	NA
269	xx Liu	Yes	35	5.63	+	904	9.91	>500.00

Table S2. Information of Cohort II: Validation test (Another clinical retrospective study).

ID	Name	Treatment	drug1	drug2	Group	CDAI	DAS28	CRP
5	xx Liu	Anti-TNF-α	Etanercept	Etanercept	NDJ	NA	NA	11.6
107	xx Zhou	Anti-IL-6	Tocilizumab	Tocilizumab	NDJ	NA	NA	1.28
112	xx Nie	Anti-TNF-α	Etanercept	Etanercept	NDJ	9	2.75	2.28
126	xx Zhu	Anti-TNF-α	Etanercept	Etanercept	NDJ	9	2.76	1.88
135	xx Gong	Anti-TNF-α	Etanercept	Etanercept	DJ	15	4.4	30.1
138	xx ZHENG	Anti-TNF-α	Etanercept	Etanercept	NDJ	NA	NA	NA
141	xx Wang	Anti-TNF-α	Etanercept	Etanercept	NDJ	10	2.89	1.58
154	xx Song	Anti-TNF-α	Etanercept	Etanercept	DJ	16	3.65	3.1
175	xx Yan	Anti-TNF-α	Etanercept	Etanercept	DJ	15	4.19	12.6
187	xx Zhong	Anti-TNF-α	Enbrel	Etanercept	NDJ	17	NA	4.71
188	xx Li	Anti-IL-6	Tocilizumab	Tocilizumab	NDJ	9	2.74	1.43
190	xx Sha	Anti-TNF-α	Ann_BNO	Etanercept	DJ	10	3.27	5.57
198	xx He	Anti-IL-6	Tocilizumab	Tocilizumab	NDJ	7	2.85	5.39
199	xx Luo	Anti-IL-6	Tocilizumab	Tocilizumab	DJ	12	3.63	15.2
202	xx Xiao	Anti-IL-6	Tocilizumab	Tocilizumab	NDJ	14	NA	1.45
204	xx Liu	Anti-TNF-α	Ann_BNO	Etanercept	NDJ	7	2.71	2.61
205	xx Guo	Anti-TNF-α	Enbrel	Etanercept	NDJ	3	1.49	1.53
215	xx Huang	Anti-TNF-α	Etanercept	Etanercept	NDJ	3	1.89	5.19
217	xx Yang	Anti-IL-6	Tocilizumab	Tocilizumab	DJ	19	4.46	16.7
218	xx Zhou	Anti-TNF-α	Ann_BNO	Etanercept	DJ	12	3.39	2.69
221	xx Wang	Anti-TNF-α	Ann_BNO	Etanercept	DJ	16	4.09	9.46
225	xx Li	Anti-TNF-α	Etanercept	Etanercept	NDJ	13	3.09	1
226	xx Chen	Anti-TNF-α	Ann_BNO	Etanercept	DJ	23	4.74	13.5
227	xx Zhu	Anti-IL-6	Tocilizumab	Tocilizumab	NDJ	6	2.79	3.7
229	xx Nie	Anti-TNF-α	Etanercept	Etanercept	DJ	13	3.61	4.33
235	xx Yu	Anti-TNF-α	Tocilizumab	Tocilizumab	NDJ	6	2.52	4.35
238	xx Luo	Anti-TNF-α	Etanercept	Etanercept	NDJ	6	2.61	3.6
239	xx Yang	Anti-IL-6	Tocilizumab	Tocilizumab	NDJ	14	NA	NA
240	xx Ni	Anti-TNF-α	Ann_BNO	Etanercept	DJ	12	3.7	NA
243	xx Chen	Anti-TNF-α	Ann_BNO	Etanercept	DJ	23	4.75	14.5
250	xx Tang	Anti-TNF-α	Ann_BNO	Etanercept	DJ	21	4.22	4.04
251	xx Li	Anti-TNF-α	Ann_BNO	Etanercept	DJ	19	3.53	3.24
253	xx Xu	Anti-TNF-α	Etanercept	Etanercept	NDJ	16	NA	9.39
258	xx Hu	Anti-TNF-α	Etanercept	Etanercept	DJ	17	3.76	NA
259	xx Tan	Anti-TNF-α	Etanercept	Etanercept	DJ	13	4.14	31
261	xx Zhang	Anti-TNF-α	Etanercept	Etanercept	DJ	18	3.93	3.09
262	xx Zhao	Anti-TNF-α	Ann_BNO	Etanercept	DJ	15	3.84	6.18
267	xx Ren	Anti-TNF-α	Ann_BNO	Etanercept	NDJ	10	NA	11.8
269	xx Liu	Anti-TNF-α	Etanercept	Etanercept	NDJ	7	2.71	2.84
273	xx Li	Anti-TNF-α	Etanercept	Etanercept	NDJ	9	2.61	NA
